# Study of the Microstructure and Mechanical Properties of Steel Grades for Ship Hull Construction

**DOI:** 10.3390/ma17235687

**Published:** 2024-11-21

**Authors:** Siavash Imanian Ghazanlou, Ahmad Mobasher Amini, Félix-Antoine Carrier, Dilip K. Sarkar, Kashif Rehman, Mousa Javidani

**Affiliations:** 1 Department of Applied Sciences, University of Québec at Chicoutimi (UQAC), 555 Boulevard de l’Université, Saguenay, QC G7H 2B1, Canada; igsiavash@etu.uqac.ca (S.I.G.);; 2 Chantier Davie Canada Inc., Lévis, QC G6V 0K4, Canada; 3 Algoma Steel Inc., 105 West St, Sault Ste. Marie, ON P6A 7B4, Canada

**Keywords:** marine steel, hot rolling, quench and tempering, thermomechanical processing, microstructure, mechanical properties

## Abstract

This paper comprehensively examines three structural steel grades’ microstructural features and mechanical properties, evaluating their suitability for shipbuilding applications. The steels analyzed include quench and tempered (Q and T) steel, thermomechanical controlled processed (TMCP) steel, and hot rolled (HR) steel. A microstructural characterization was performed using optical microscopy (OM), scanning electron microscopy (SEM), and electron backscatter diffraction (EBSD). The analysis was complemented by extensive mechanical testing including assessments of hardness, tensile, and Charpy impact tests across a range of temperatures. Additionally, corrosion behavior was evaluated using the potentiodynamic polarization test. The findings revealed that Q and T grade steel exhibited the most refined microstructure, characterized by a complex mixture of ferrite, tempered martensite, upper bainite, and Fe_3_C phases. In contrast, the TMCP grade steel demonstrated a balanced microstructure of polygonal ferrite and pearlite. Meanwhile, the HR grade steel contained polygonal ferrite and aligned pearlite. The tensile testing results demonstrated that the Q and T grade steel had superior hardness, yield strength (YS), and ultimate tensile strength (UTS), although it exhibited the lowest elongation % (El %). The TMCP grade steel met all ABS standards for marine steels, displaying optimal YS, UTS, and El %. Despite the superior YS of the HR grade steel, it did not meet the necessary criteria for UTS. Charpy impact tests revealed that the TMCP grade steel exhibited the highest impact energy absorption across a range of temperatures. As a result, the TMCP grade steel emerged as the optimal choice for ship construction, fulfilling all ABS requirements with a balanced combination of strength, ductility, and impact energy absorption. Additionally, the potentiodynamic polarization results revealed that the Q and T grade steel demonstrated the highest corrosion resistance. Following Q and T steel, the HR grade steel ranked second in corrosion resistance, with TMCP steel closely behind, showing only a slight difference.

## 1. Introduction

The selection of steel is crucial for ship hull construction, as it ensures the vessel’s structural integrity, durability, and resistance to harsh marine conditions. Commonly used steel grades in shipbuilding include A, B, D, E, AH, DH, EH, and FH, with their chemical compositions detailed in Table 1 [1].

Among these, AH, EH, and FH are particularly suited for ship construction due to their higher yield strength and impact energy at sub-zero temperatures. The impact energy of steel is highly sensitive to temperature. At lower temperatures, the ability of steel to absorb impact energy reduces. Conversely, at higher temperatures, this ability increases. The sharp transition from ductile to brittle behavior is known as the ductile-to-brittle transition temperature, which is critical when selecting steel grades for ship construction. Operating steel grades below their ductile-to-brittle transition temperature has historically led to ship failures [2,3,4].

HT processes significantly enhance the microstructure and mechanical properties of steel grades. Several HT techniques have been employed to improve steel performance [5,6,7,8]. The Q and T technique, in particular, has demonstrated success in enhancing the impact energy of EH47 steel, increasing it from 64 J to 107.5 J at −40 °C, due to the formation of a well-balanced microstructure of acicular ferrite, polygonal ferrite, and tempered martensite [8]. This improvement aligns with studies that highlight the positive effects of acicular ferrite on the impact energy absorption of steel at sub-zero temperatures [9,10].

The improvement in properties achieved through the TMCP method in shipbuilding steels surpasses that of traditional rolling methods [11,12]. For EH40, the TMCP program includes annealing, rolling through both recrystallization and non-recrystallization zones, followed by rapid cooling to ambient temperature (referred to as the “Two-Rolling TMCP” method). This process resulted in the formation of polygonal or quasi-polygonal ferrites, along with a small amount of pearlite. This microstructure contributed to ductile fracture behavior during Charpy impact tests at sub-zero temperatures [13]. In the case of EH36 steel, the Two-Rolling TMCP method resulted in the formation of acicular ferrite, polygonal ferrite, grain boundary ferrite, and pearlite. This microstructure exhibited high fracture toughness and demonstrated ductile behavior under fatigue [14]. For the EH47, the Two-Rolling TMCP method significantly improved its yield strength (from 520 to 670 MPa), ultimate tensile strength (from 774 to 829 MPa), and elongation (from 21.3 to 26.3%) at −130 °C. This enhancement can be attributed to acicular ferrite (AF) formation, a low amount of granular bainite, and a high-volume fraction of large polygonal ferrite [15]. The application of the Two-Rolling TMCP method to both EH36 and FH36 resulted in enhanced microstructures, leading to a more significant improvement in strength, impact energy, and fracture toughness compared to the traditional TMCP method applied to E36 [16]. Numerous reports have also underscored the positive effects of this Two-Rolling TMCP method on the microstructure and mechanical properties of EH36 [14,16], FH36 [16], EH40 [13], and EH47 [15].

Shipbuilding steels must withstand the forces of waves, substantial bending moments caused by surging waters, temperature fluctuations from cold winters to hot summers, as well as seawater corrosion. Therefore, in addition to having adequate mechanical properties, hull steel must exhibit excellent corrosion resistance. Corrosion can weaken the ship’s structural integrity, reduce its service life, jeopardize navigation safety, and damage operational performance. Consequently, assessing the corrosion of hull structure steel has always been significant in ship design and construction [17,18,19,20].

This study offers a comprehensive analysis of the microstructure and mechanical properties of three steel grades. The primary objective is to assess whether these steel grades meet the mechanical property requirements set by the American Bureau of Shipping (ABS) standard for ship construction. A detailed microstructural characterization was carried out using OM, SEM, and EBSD, alongside mechanical testing, including hardness, tensile, and Charpy impact tests over a range of temperatures. Additionally, the corrosion behavior of the steel samples was investigated. This work not only deepens our understanding of the microstructural evolution in shipbuilding steels but also provides valuable insights for optimizing steel properties to enhance performance and durability in marine environments.

## 2. Experimental Procedure

### 2.1. The Chemical Composition of the Steel Grades

This report provides an in-depth analysis of the microstructural and mechanical properties of Q and T, TMCP (EH36), and HR (CSA G40.21 grade 60 W) steel grades. Table 2 illustrates the chemical composition of the steel grades.

### 2.2. The Steel Fabrication Processes

During the HR process, the billet first underwent homogenization through a heat treatment at 1200 °C for 4 h. Following this, the billet was subjected to the rolling process. The finishing rolling temperature for the austenite recrystallized region was set at 1050 °C. This temperature was chosen to facilitate optimal deformation and grain refinement. After rolling, the steel was cooled in the air [21]. In the Q and T process, the sample was subjected to annealing at a temperature of 900 °C for 30 min. The goal of this annealing process was to induce a transformation in the microstructure, converting it into austenite and polygonal ferrite. Subsequently, rapid water quenching was followed, facilitating the transformation of austenite into martensite. Following quenching, tempering was carried out at 620 °C for 30 min [8]. The purpose of tempering was to further modify the microstructure via tempering the martensite. Finally, the sample was removed from the heat source and allowed to cool naturally in ambient air. In the Two-Rolling TMCP method, the process initiated with a homogenization stage, exposing the billet to a temperature of 1200 °C for 4 h. Following this, the billet is subjected to rolling in two distinct regions: the recrystallized and non-recrystallized zones. The finishing rolling temperature for the recrystallized region was set at 1050 °C while the finish rolling temperature for the non-recrystallized region was set at 850 °C. After the rolling, the steel plate was subjected to air-cooling to 780 °C at a rate of 5 °C/s, followed by immediate water quenching until reaching ambient temperature [15]. These temperature and time parameters were selected in industrial practice based on established metallurgical principles and practical experience, aiming to achieve the desired microstructural characteristics and mechanical properties for each process.

### 2.3. The Microstructure Characterization Methods

For OM, SEM, and EBSD analysis, the samples were prepared using the standard grinding and polishing method. After preparing, the samples were etched with a 3% Nital solution for OM imaging (Nikon Eclipse ME600, Nikon Corporation, Tokyo, Japan) and SEM imaging (JEOL JSM-6480LV, JEOL USA, Inc., Peabody, MA, USA). For EBSD analysis, a non-etched, fully polished sample was used. The EBSD analysis was conducted on an SEM equipped with an HKL Nordlys EBSD detector to examine the microstructure of the rolling direction–normal direction (RD–ND) sections. EBSD scans were conducted with a step size of 1.5 μm, ensuring an indexing rate greater than 0.7 for all samples. If the indexing rate was below this threshold, the entire sample preparation and EBSD measurement process were repeated. The EBSD patterns were analyzed using the ATEX© analysis software package (version 4.14). Post-processing, including “correction-noise reduction”, was performed in a single step after the EBSD measurement.

### 2.4. The Evaluation Procedures for Mechanical Properties

In the next step, the mechanical properties were studied. A microhardness test was performed based on the Vickers hardness at a load of 50 N at 20 s and an average of 10 readings was reported. The tensile test was conducted according to ASTM E8 standards [22] using a sub-size specimen with an overall length of 100 mm at a strain rate of 4.7 × 10^−4^ (a cross-head speed of 0.5 mm/min) using the MTS 810 System and an average of 3 repetitions was reported for each sample. The Charpy V-notch impact test was performed based on the sub-size specimens (10 mm × 5 mm × 55 mm) according to the ASTM A370 standard [23] at room temperature, 0, −20, and −40 °C using an Instron impact-testing machine DI-300. An average of three readings was reported for each sample at each temperature.

### 2.5. Corrosion Resistance Measurement Method

Electrochemical experiments were conducted using a PGZ100 potentiostat and a 300 cm^3^ EG and G PAR flat cell from London Scientific in London, ON, Canada, employing a three-electrode system. The corrosion cell kit included an Ag/AgCl reference electrode, a platinum auxiliary electrode, and the steel surface as the working electrode. Polarization tests were performed in a 3.5 wt% NaCl solution with a pH of 5.9 at room temperature by incrementally adjusting the potential. The open circuit potential (OCP) was measured for 30 min before performing the polarization process.

## 3. Results and Discussions

### 3.1. Microstructure Characterization

**Figure 1:**
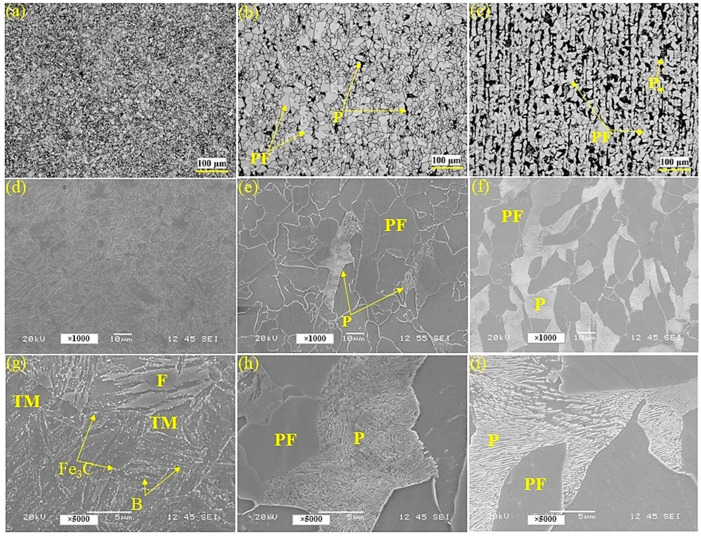
The OM (**a**–**c**) and SEM (**d**–**f**) images of the Q and T (**a**,**d**,**g**), TMCP (**b**,**e**,**h**), and HR (**c**,**f**,**i**) steel grades.

Figure 1 presents the OM and SEM images of the steel samples. The Q and T sample exhibits the finest microstructure, characterized by a complex mixture of ferrite (F), tempered martensite (TM), upper bainite (B), and Fe_3_C phases. The refinement of the microstructure in the Q and T sample can be attributed to the controlled annealing, cooling, and subsequent tempering process, which promotes the formation of fine-grained structures and can lead to increased strength. The TMCP and HR samples predominantly exhibit a microstructure composed of polygonal ferrite (PF) and pearlite (P). However, the fraction of pearlite in the TMCP sample is considerably lower compared to the HR grade. Furthermore, in the HR sample, the pearlite phase demonstrates alignment with the rolling direction (RD), indicating an anisotropic microstructure. As a result, the TMCP sample may exhibit higher ductility than the HR sample, while the HR sample can demonstrate greater strength compared to the TMCP sample.

**Figure 2:**
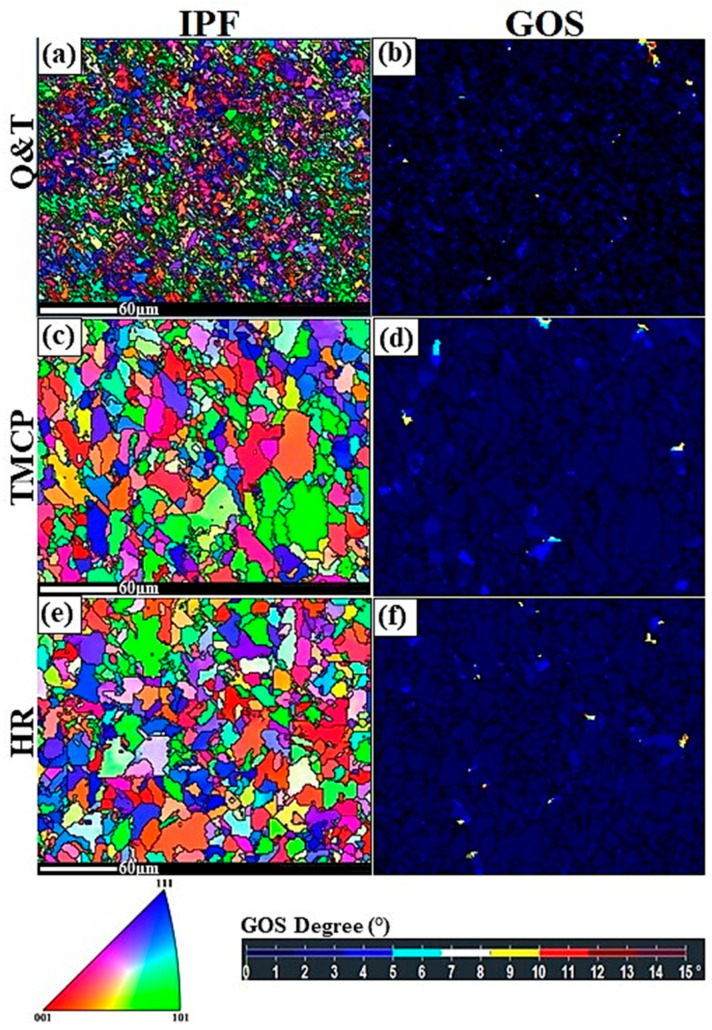
The inverse pole figure (IPF = (**a**) Q and T, (**c**) TMCP, and (**e**) HR) maps on the left and grain orientation spread (GOS = (**b**) Q and T, (**d**) TMCP, and (**f**) HR) maps on the right from the steel grades.

Figure 2 shows the inverse pole figure (IPF) maps and grain orientation spread (GOS) maps from the steel grades. As depicted in IPF maps, the Q and T sample exhibits the finest microstructure among the three with an average grain size of approximately 4 μm, whereas the TMCP and HR samples have average grain sizes of around 15.5 μm and 14 μm, respectively. Furthermore, the majority of grains in all grades have an equiaxed morphology, indicating a fully recrystallized microstructure. Additionally, the analysis of grain orientation spread (GOS) maps indicates that most grains (85% in all grades) have a GOS value < 2° (the black areas in GOS maps). A GOS value < 2° is indicative of a recrystallized grain [24,25,26]. This observation aligns with the findings from the IPF maps, confirming the presence of predominantly equiaxed recrystallized grains. These results demonstrate that the designed HT, TMCP, and Q and T processes have successfully led to the formation of a mostly recrystallized microstructure across all grades of steel.

**Figure 3:**
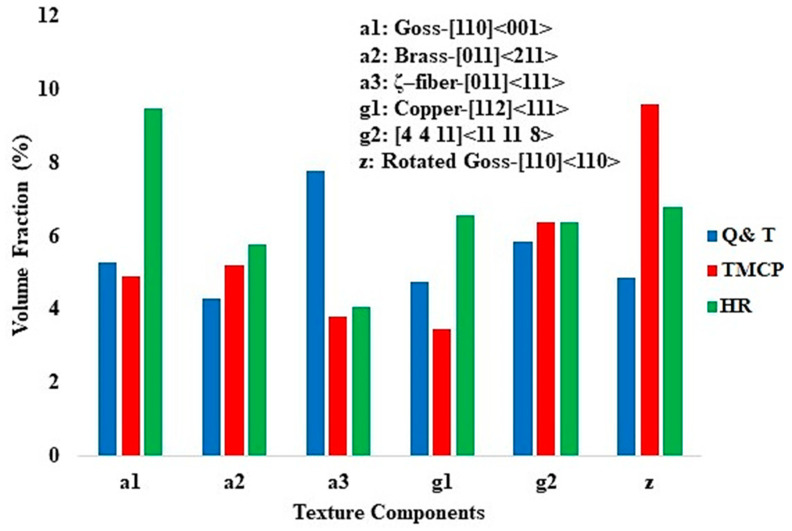
The texture components of the steel grades.

Figure 3 shows the texture components diagrams for the steel grades. This figure provides valuable information regarding the texture components created within steel grades during the fabrication processes. The calculation of the texture components was carried out under similar conditions to those used for calculating the IPF maps of the normal direction (ND)–rolling direction (RD) sections for the composites, as shown in Figure 2. Based on Figure 3, the application of the HR, HT, and TMCP methods resulted in the formation of different texture components with varying intensities (vol.%) in the steel samples. These texture components include Goss-[110]<001> [27], Brass-[011]<211> [28], rotated Goss-[110]<110> [29], ζ–fiber (fiber axis <001>, parallel to the normal direction-[011]<111> [28]), Copper-[112]<111> [30], and [4 4 11]<11 11 8>. The [4 4 11]<11 11 8> component reflects the profile of the shear strain, which arises due to the influence of temperature on the flow stress and the through-thickness profile of the Zener-Hollomon parameter [28]. Brass and copper typically exhibit a deformed microstructure; however, the presence of Goss and rotated Goss textures are indicative of a recrystallized microstructure [25,31,32]. As shown in the Q and T grade steel, the two main texture components are ζ–fiber and [4 4 11]<11 11 8>. The ζ–fiber usually can be seen in hot rolled steels and [4 4 11]<11 11 8> is a type of shear texture [28]. The presence of these two types of textures in the Q and T grade steel can be attributed to the quenching and tempering process that led to the formation of tempered martensite (TM). The martensite, which formed via a shear transformation, likely contributed to the development of these texture components, particularly the [4 4 11]<11 11 8> shear texture. In the TMCP steel, the two primary texture components are rotated Goss and [4 4 11]<11 11 8> shear texture. The presence of rotated Goss indicates the formation of a mainly recrystallized microstructure, which is consistent with the GOS maps in Figure 2. The [4 4 11]<11 11 8> shear texture arises from the application of two rolling processes conducted above and below the austenite recrystallization temperature. In the HR steel, the three prominent texture components are Goss, rotated Goss, and copper. The presence of Goss and rotated Goss indicates the formation of a mostly recrystallized microstructure, which aligns with the GOS maps in Figure 2. The formation of copper texture signifies the existence of a partially deformed microstructure created during the hot rolling of this steel. As illustrated in Figure 2, a small fraction of the microstructure remains non-recrystallized (with GOS values > 2°).

### 3.2. Mechanical Properties

#### 3.2.1. Hardness and Tensile Properties and Fracture Mechanisms

**Figure 4:**
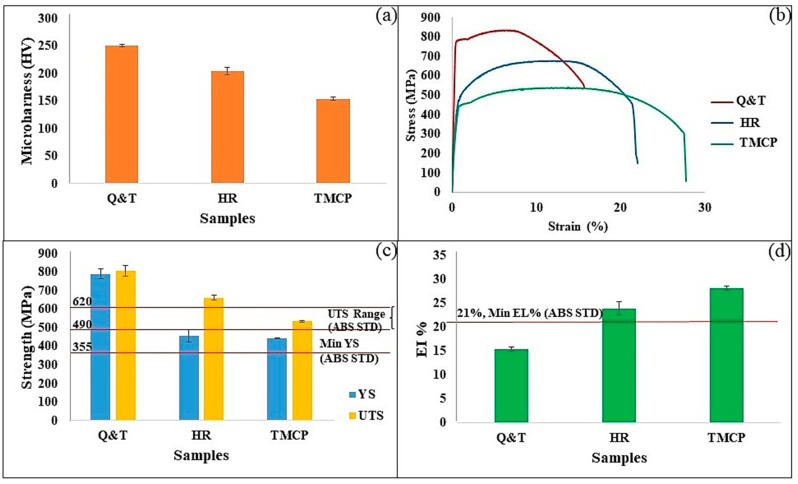
The hardness (**a**), stress–strain diagrams (**b**), YS and UTS values (**c**), and El% (**d**) of the samples.

Figure 4 depicts hardness, stress–strain diagrams, YS, UTS, and El% values for the Q and T, TMCP, and HR steel samples. Moreover, this figure includes the mechanical property thresholds specified by ABS for EH36 marine steels. This comparative analysis aims to ascertain which among the Q and T, TMCP, and HR grades meet the specified mechanical property requirements identified by ABS [21]. As depicted, Q and T steel exhibits the highest levels of hardness, YS, and UTS among the considered steel grades, while concurrently displaying the lowest El% values compared to TMCP and HR steel. These differences can be attributed to their microstructural characteristics. The finer microstructure of Q and T steel, enriched with hard phases and fine Fe_3_C dispersion, contributes significantly to its elevated hardness and strength values, albeit at the expense of ductility. Conversely, HR and TMCP steel, characterized by the presence of polygonal ferrite and pearlite phases, exhibit comparatively lower levels of hardness and strength but demonstrate improved ductility. Furthermore, HR steel outperforms TMCP steel in terms of hardness, YS, and UTS, albeit exhibiting reduced ductility because of the higher fraction of the hard pearlite phase in the HR grade. Regarding ABS requirements, Figure 4c indicates that all steel grades surpass the minimum YS stipulated by ABS. However, when considering the UTS range mandated by ABS (490 to 620 MPa), only the TMCP grade falls within this specified range. Q and T and HR steel exceed the upper limit of the UTS range, departing them from the specified range. Furthermore, as shown in Figure 4d, both TMCP and HR steel exceed the minimum El% requirement of 21% set by ABS, demonstrating values above this threshold. The Q and T steel falls below the minimum El% requirement, placing it outside the specified range. Therefore, based on the ABS tensile property criteria for EH36 marine steels, only the TMCP grade meets all the required thresholds.

**Figure 5:**
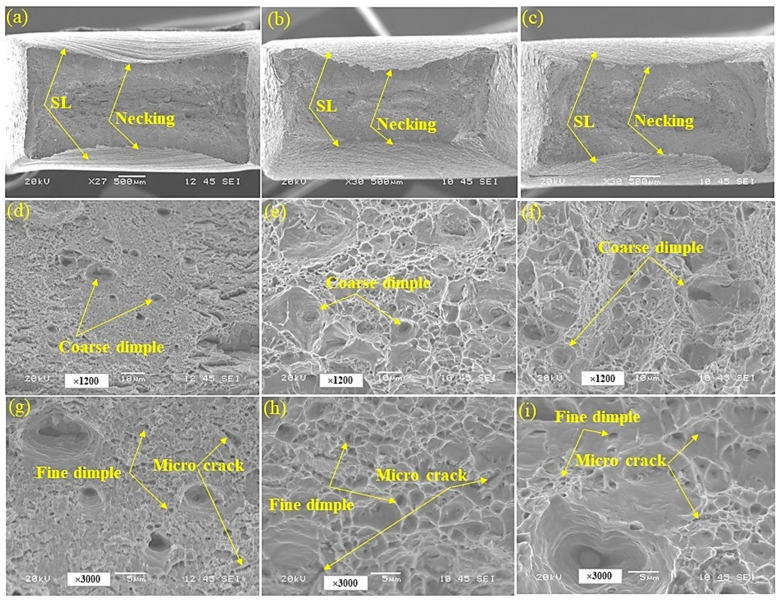
The SEM images from the fracture surface of the Q and T (**a**,**d**,**g**), TMCP (**b**,**e**,**h**), and HR (**c**,**f**,**i**) steel grades.

Figure 5 shows the SEM images from the fracture surface of tensile-tested steel grades. As illustrated in Figure 5a–c, all of the samples underwent necking before fracture and some slip lines (SL) can be identified as signs of ductile fracture. As illustrated in Figure 5d–f, some coarse dimples can be seen in all of the samples. Dimples are characteristic of a ductile fracture, indicating that the material undergoes substantial plastic deformation before failure. These rounded, cup-shaped dimples form on the fracture surface as a result of the nucleation, growth, and the coalescence of microvoids during deformation [33,34,35,36]. A comparison of Figure 5d with Figure 5e,f reveals that the fracture surface of Q and T steel contains significantly fewer coarse dimples than those observed in TMCP and HR steel. This indicates that Q and T steel underwent a less plastic deformation before fracturing compared to TMCP and HR steel. The fewer coarse dimples in Q and T steel suggest a lower degree of plastic deformation before fracture, which aligns with its higher strength and reduced ductility. In contrast, the greater number of coarse dimples in TMCP and HR steel indicates a more extensive plastic deformation before fracture, consistent with their lower strength and higher ductility. Figure 5g–i also reveal the presence of fine dimples (microvoids) and micro-cracks on the fracture surfaces of all samples. Microvoids and micro-cracks play a critical role in the ductile fracture process during tensile tests. As the material is subjected to tensile stress, microvoids nucleate, then they grow and coalesce with continued deformation, leading to the formation of larger cavities. The coalescence of microvoids forms micro-cracks, which propagate and create fracture paths, ultimately resulting in material failure [37,38]. This process highlights the ductile nature of the fracture, where considerable plastic deformation occurs before the material failure.

#### 3.2.2. Charpy Impact Test

**Figure 6:**
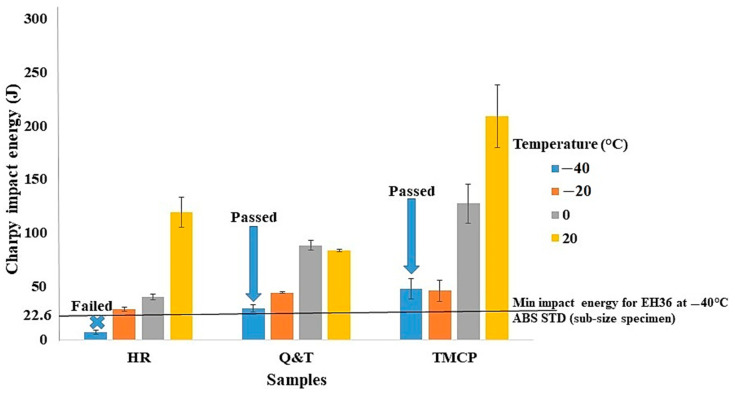
The average impact energy of Q and T, TMCP, and HR steel grades as a function of temperature.

Figure 6 presents the average Charpy impact energy of Q and T, TMCP, and HR steel grades across varying temperatures. As depicted, a notable decrease in Charpy impact energy is observed with decreasing temperature. This decline signifies a reduced capacity of the steel to absorb energy before fracture. Conversely, at elevated temperatures, steels tend to exhibit more ductile behavior, characterized by enhanced energy absorption before fracturing. The observed transition from ductile to brittle behavior as temperature decreases is attributed to an increased susceptibility to brittle fracture mechanisms such as cleavage. This phenomenon is commonly known as the ductile-to-brittle transition [39,40]. In addition, among the steel grades, TMCP steel consistently exhibits the highest impact energy values across the temperature range. Following TMCP steel, Q and T steel ranks second in impact energy. However, HR steel initially surpasses Q and T steel in impact energy at 20 °C. Nevertheless, as the temperature decreases, HR steel demonstrates the lowest impact energy among the steel grades. In TMCP steel, a high fraction of the polygonal ferrite phase provides a ductile matrix, further enhancing the steel’s impact energy. Additionally, the presence of pearlite contributes to its toughness by inhibiting crack propagation. Another study indicated that the presence of polygonal ferrite, among other ferrite morphologies, maintains the impact energy of pipeline steel at sub-zero temperatures [41]. Moreover, other studies have suggested that in ferrite–pearlite steels, the presence of pearlite can decrease the rate of crack propagation through the crack deflection mechanism [42,43,44,45]. As a result, TMCP steel consistently exhibits the highest impact energy values across the temperature range due to its favorable microstructural characteristics for energy absorption. In Q and T steel, the existence of a fine microstructure and the presence of multiple phases contribute to energy absorption during impact loading, resulting in relatively high impact energy values. In addition, the uniform dispersion of Fe_3_C within the matrix can act as a barrier in the crack propagation path [46,47]. However, Q and T steel ranks slightly lower in impact energy compared to TMCP steel due to the presence of hard phases such as tempered martensite and bainite. In HR grade steel, the reduced ferrite content lowers its ability to absorb impact energy compared to TMCP grade steel. Furthermore, the alignment of the pearlite phase with the rolling direction in HR grade steel can create localized stress concentrations, which reduces toughness in certain orientations, especially at lower temperatures. This alignment can hinder energy absorption and promote crack propagation within the steel matrix. Consequently, HR grade steel exhibits the lowest impact energy among the steel grades. Additionally, Figure 6 illustrates a comparison between the average impact energies of TMCP, Q and T, and HR steel grades and the minimum impact energy requirement for EH36 at −40 °C, as specified by the ABS standard [21]. As explained previously, the Charpy V-notch impact test was performed on sub-size specimens. Therefore, the results should be compared to two-thirds of the standard impact energy value as specified by the ABS standards [21]. This adjustment ensures a proportional comparison between the energy absorbed by the sub-size specimen and the full-size standard specimen, providing an accurate evaluation of the material’s impact energy under similar conditions. As shown at −40 °C, TMCP and Q and T steel surpass the minimum impact energy requirements for EH36 steel, while HR steel falls short, indicating its unsuitability for use at this temperature. Consequently, according to the tensile and Charpy impact energy results depicted in Figure 4 and Figure 6, respectively, only TMCP steel meets all of the ABS benchmarks, making it the most promising option for constructing ship hulls among all grades.

### 3.3. Corrosion Test

**Figure 7:**
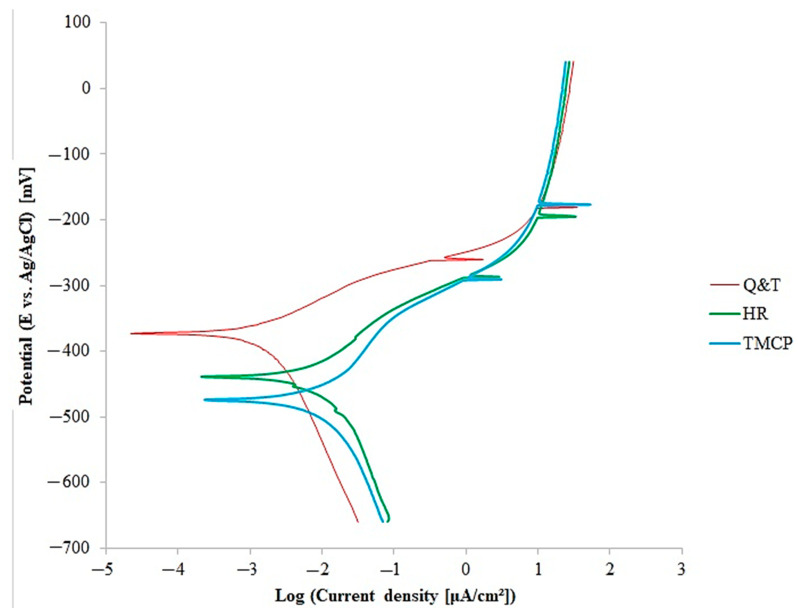
Tafel polarization plots for the steel samples in a 3.5 wt% NaCl solution (pH ≈ 5.9) at room temperature.

The potentiodynamic polarization (Tafel) curves were utilized to analyze the corrosion behavior of the steel grades. Figure 7 displays the Tafel polarization plots for the steel samples in a 3.5 wt% NaCl solution (pH ≈ 5.9) at room temperature. Table 3 presents the electrochemical parameters derived from the potentiodynamic polarization diagrams using the Tafel extrapolation method. According to Figure 7 and Table 3, the Q and T grade exhibits the lowest corrosion current, the highest polarization resistance, and the lowest corrosion rate among the steel samples. Following Q and T steel, HR steel ranks second, followed by TMCP steel in third place with a slight difference. Consequently, Q and T steel demonstrates the highest corrosion resistance. The variation in corrosion resistance can be attributed to both the chemical composition and microstructural features. According to Table 2, Q and T steel contains the highest concentrations of Cr and Mo, which are known to inhibit pitting corrosion by facilitating the formation of a protective Cr_2_O_3_-MoO_3_ passive layer on the substrate surface in corrosive environments [48,49]. Furthermore, a homogeneous dispersion of Fe_3_C throughout the Q and T matrix serves as a barrier against corrosive solution penetration by effectively filling pores. Flaws and surface imperfections in metals typically serve as active sites for corrosion. The particles embedded in these voids, particularly those capable of effectively filling them [50,51], contribute to enhancing the corrosion resistance of the sample. The chemical composition and microstructural characteristics of TMCP and HR steel were similar, resulting in no big difference between their corrosion resistances, but the corrosion resistance of HR steel was slightly higher. During the Q and T process, especially in the tempering phase, significant stress relief occurs, resulting in the Q and T grade having the highest corrosion resistance. Residual stresses can act as stress concentrators, increasing the risk of pitting in corrosive environments [52,53]. Relieving these stresses reduces the likelihood of localized corrosion. On the other hand, TMCP steel undergoes two rolling cycles, one below the austenite recrystallization temperature, causing higher residual stress compared to HR steel, which undergoes a single cycle above this temperature. Consequently, HR steel shows higher corrosion resistance than TMCP steel.

## 4. Conclusions

This study offers an in-depth analysis of the microstructural and mechanical properties of three steel grades with potential applications in shipbuilding. The microstructures of Q and T, TMCP, and HR steels were thoroughly characterized using OM, SEM, and EBSD analyses. These analyses were complemented by evaluations of mechanical properties through hardness, tensile, and Charpy impact tests across a range of temperatures. The main results are as follows:

- The microstructural investigations revealed that Q and T steel exhibited the finest microstructure, characterized by a complex mixture of ferrite, tempered martensite, upper bainite, and Fe3C phases. TMCP steel demonstrated a balanced microstructure with polygonal ferrite and pearlite. HR displayed an anisotropic microstructure with polygonal ferrite and aligned pearlite.
- The texture analysis highlighted that Q and T steel contained significant ζ-fiber and [4 4 11]<11 11 8> shear textures, indicative of its quenched and tempered nature. TMCP steel displayed a predominance of rotated Goss and [4 4 11]<11 11 8> shear textures, consistent with its thermomechanical processing, while HR steel showed Goss, rotated Goss, and copper textures, reflecting its hot rolled processing history.
- Tensile testing revealed that Q and T steel exhibited the highest hardness, YS, and UTS but the lowest El% among the steel grades. TMCP steel demonstrated tensile properties that met all the ABS standards for marine steels, including optimal YS, UTS, and El%, making it the most suitable for shipbuilding applications. HR steel, while showing superior strength, did not fully meet the required criteria for the UTS range. The fracture surface analysis via SEM further verified these findings, showing fewer coarse dimples and microvoids in Q and T steel, indicative of its lower ductility and higher strength, whereas TMCP and HR steel exhibited coarser dimples, aligning with their higher ductility.
- As temperatures dropped, the impact energy of all the steel grades decreased, indicating a reduced ability to absorb energy before fracturing. TMCP steel consistently exhibited the highest impact energy across the temperatures due to its favorable microstructure, which includes a significant amount of ductile polygonal ferrite and pearlite that help resist crack propagation. Q and T steel ranked second, with its fine microstructure aiding in energy absorption, although the presence of harder phases like tempered martensite and bainite slightly reduced its impact energy. HR steel did not perform well at sub-zero temperatures, due to its anisotropic microstructure and lower ferrite content. Among the grades, only TMCP steel fully meets the ABS standard requirements for tensile and impact energy properties for EH36 grade, making it the most suitable grade for ship hull construction.
- The potentiodynamic polarization curves showed that Q and T steel demonstrated superior corrosion resistance with the lowest corrosion current and rate, attributed to its higher Cr and Mo content, which helps form a protective oxide layer. Its uniform Fe3C dispersion further blocked corrosion pathways. In addition, during tempering, stress relief further minimizes corrosion risks by reducing residual stresses, which can act as corrosion initiation sites. In contrast, the TMCP grade, with its two rolling cycles, retains higher residual stresses, lowering its corrosion resistance compared to HR steel, which undergoes a less stressful single rolling cycle.
- Consequently, this study underscores the critical role of processing techniques in tailoring the microstructure and mechanical properties of shipbuilding steel grades. Among the steel grades, TMCP grade steel emerged as the optimal choice for shipbuilding applications due to its balanced combination of strength, ductility, and impact energy, meeting all ABS requirements. These insights provide valuable guidelines for the selection and optimization of steel grades for enhanced performance and durability in marine environments, ensuring the safety and efficiency of vessel operation.

## Figures and Tables

**Table 1:** The chemical composition of the some shipbuilding steel (wt%) [1].

Steel Grade	C	Mn	P	S	Si	Ni	Cr	Mo	Cu
A	0.21 max	2.5 × C min	0.035 max	0.035 max	0.5 max	-	-	-	-
B	0.21 max	0.8 max	0.035 max	0.035 max	0.35 max	-	-	-	-
D	0.21 max	0.60–0.90	0.035 max	0.035 max	0.10–0.35	-	-	-	-
E	0.18 max	0.70–0.90	0.035 max	0.035 max	0.10–0.35	-	-	-	-
AH/DH/EH	0.18 max	0.90–1.6	0.035 max	0.035 max	0.1–0.5	0.4 max	0.2 max	0.08 max	0.35 max
FH	0.16 max	0.90–1.6	0.025 max	0.025 max	0.1–0.5	0.8 max	0.2 max	0.08 max	0.35 max

**Table 2:** The chemical composition of the steel grades (wt.%) (Fe: Bal.).

Element/Steel Grade	C	Mn	P	S	Si	Cr	Ni	Cu	Mo	Al	Nb	V	B	Ti
Q and T	0.18	1.25	0.011	0.002	0.19	0.12	0.02	0.03	0.120	0.025	0.000	0.007	0.0021	0.048
TMCP	0.05	1.37	0.009	0.002	0.31	0.08	0.08	0.08	0.060	0.033	0.038	0.043	0.0001	0.002
HR	0.19	1.50	0.012	0.000	0.31	0.02	0.02	0.02	0.000	0.032	0.009	0.062	0.0001	0.002

**Table 3:** Electrochemical parameters derived from the potentiodynamic polarization diagrams using the Tafel extrapolation method.

Sample	β_a_ (mV/dec)	β_c_ (mV/dec)	E_corr_ (mV vs. Ag/AgCl)	I_corr_ (μA/cm^2^)	R_p_ (kΩ·cm^2^)	Corrosion Rate (µm/year)
Q and T	54	−115	−337	1.04	12.90	11
HR	106	−148	−443	8.13	2.80	88
TMCP	131	−189	−478	10.39	2.63	113

## Data Availability

The original contributions presented in the study are included in the article, further inquiries can be directed to the corresponding author.
